# Roles of Type VI Secretion System in Transport of Metal Ions

**DOI:** 10.3389/fmicb.2021.756136

**Published:** 2021-11-05

**Authors:** Xiaobing Yang, Hai Liu, Yanxiong Zhang, Xihui Shen

**Affiliations:** ^1^College of Applied Engineering, Henan University of Science and Technology (HAUST), Sanmenxia, China; ^2^Medical College, Sanmenxia Vocational Technical School, Sanmenxia, China; ^3^Qingyang Longfeng Sponge City Construction Management & Operation Co., Ltd, Qingyang, China; ^4^State Key Laboratory of Crop Stress Biology for Arid Areas, Shaanxi Key Laboratory of Agricultural and Environmental Microbiology, College of Life Sciences, Northwest A&F University, Xianyang, China

**Keywords:** type VI secretion system, effectors, metal ions, transport, regulation

## Abstract

The type VI secretion system (T6SS) is a transmembrane protein nanomachine employed by many gram-negative bacteria to directly translocate effectors into adjacent cells or the extracellular milieu, showing multiple functions in both interbacterial competition and bacteria-host interactions. Metal ion transport is a newly discovered T6SS function. This review summarizes the identified T6SS functions and highlights the features of metal ion transport mediated by T6SS and discusses its regulation.

## Introduction

The type VI secretion system (T6SS) is a transmembrane protein nanomachine employed by many gram-negative bacteria to translocate effectors directly into adjacent target cells or the extracellular milieu (Cianfanelli et al., 2016). T6SS was regarded as virulence-associated secretion apparatus because of its association with pathogenicity (Mougous et al., 2006; Pukatzki et al., 2006). However, subsequent studies have demonstrated T6SS function is involved in multiple physiological and biochemical processes apart from bacterial pathogenesis, such as interbacterial competition (Hood et al., 2010; Chassaing and Cascales, 2018), commensalism or symbiosis (Chow and Mazmanian, 2010), stress response (Weber et al., 2009; Wan et al., 2017), biofilm formation (Zhang et al., 2011; Gallique et al., 2017), and horizontal gene transfer (Borgeaud et al., 2015).

It has been reported that the T6SS function is determined by the loading effectors that can be delivered extracellularly based on energy consumption and load transport (Cianfanelli et al., 2016; Coulthurst, 2019). Many T6SS effectors related to virulence or competition that target the cell wall, membranes, and nucleic acids have been reported (Yang et al., 2018; Song et al., 2021). Several effectors with special activities have also been found. For example, a T6SS dependent effector, YezP, has been reported to combine with Zn^2+^ and contribute to Zn^2+^ transport to deal with environmental stresses (Wang et al., 2015), and subsequent studies confirmed the function of T6SS dependent transport of metal ions (Lin et al., 2017; Si et al., 2017b). This review highlights the features of T6SS-dependent metal ion transport and its regulation.

## Type VI Secretion System Functions for Metal Ions Transport

Metal ions are commonly found in all organisms in association with proteins, such as enzymes, storage proteins, and transcription factors. The metal ions are involved in many crucial biological processes and are necessary for cell survival (Hood and Skaar, 2012). Bacteria have evolved sophisticated acquisition systems, including low- and high-affinity transport systems for scavenging essential chelated or free metals from the environment (Porcheron et al., 2013). As a versatile secretion system widely distributed in Gram-negative bacteria, The T6SS was found to participate in the transport of iron, zinc, copper, manganese, and molybdate, summarized in Table 1.

**TABLE 1:** T6SS dependent ions transport related factors.

Bacteria species	T6SS effector	Membrane transporter	Metal ions	Citation
*Y. pseudotuberculosis*	YezP		Zinc	Wang et al., 2015
*Y. pseudotuberculosis*	TssS		Manganese	Zhu et al., 2021
*B. thailandensis*	TseM	MnoT	Manganese	Si et al., 2017b
*B. thailandensis*	TseZ	HmuR	Zinc	Si et al., 2017a
*B. pseudomallei*	TseZ	BhuR	Zinc	DeShazer, 2019
*B. pseudomallei*	TseM	MnoT	Manganese	DeShazer, 2019
*P. aeruginosa*	TseF	FptA/OprF	Iron	Lin et al., 2017
*P. aeruginosa*	Azu	OprC	Copper	Han et al., 2019
*P. aeruginosa*	ModA	IcmP	Molybdate	Wang et al., 2021
*C. necator*	TeoL	CubA/CstR	Iron	Li et al., 2021

### Zinc

Zinc is the second most important transition metal ion in living organisms after iron, playing an essential catalytic and structural role in several proteins involved in DNA replication, glycolysis, pH regulation, amino acid biosynthesis, extracellular peptidoglycan, and low molecular weight thiols (Porcheron et al., 2013). Zinc status is linked to the maintenance of intracellular redox buffering (Andreini et al., 2006). Both the high-affinity transporter ZnuACB and the low-affinity uptake system ZupT mediate zinc uptake across the cytoplasmic membrane (Hantke, 2005). Zinc is an essential nutrient for cells; Excess of zinc is toxic. Therefore, bacterial cells should achieve a delicate balance between ensuring sufficient zinc concentrations to fulfill essential functions while limiting concentration to prevent toxic effects. Zinc homeostasis is mediated by a network of zinc influx and efflux pumps (Wang et al., 2012; Wang and Fierke, 2013).

Wang et al. (2015) reported that the T6SS-4 from *Yersinia pseudotuberculosis* (*Yptb*) can combat multiple adverse stresses and host nutritional immunity, by displaying an unexpected function in transport of Zn^2+^. Zinc transport is achieved by T6SS-4-mediated secretion of a Zn^2+^-binding protein substrate, YezP (YPK_3549), which binds Zn^2+^ with high affinity, and represents a novel class of T6SS effector distinct from those extensively studied as bacteriolytic toxins or eukaryotic cell-targeting effectors. Hydroxyl radicals are deleterious reactive oxygen species that are often generated via Fenton chemistry under stress conditions (Mols and Abee, 2011). T6SS-4 was critical to neutralize hydroxyl radicals accumulated under adverse stress conditions, by accumulating Zn^2+^, which can mitigate hydroxyl radicals to reduce them damage. By mitigating the detrimental hydroxyl radicals induced by multiple stresses, T6SS-4 provided a molecular explanation to the phenomenon of “cross-protection” in which cells subjected to one stress become resistant to distinctly different insults (Isohanni et al., 2013). Consistent with the function of T6SS-4 in combating stress, its expression is regulated by multiple transcription regulators, such as OmpR (Gueguen et al., 2013; Zhang et al., 2013), OxyR (Wang et al., 2015), RovM (Song et al., 2015), RpoS (Guan et al., 2015), and RelA (Yang et al., 2019), all of which respond to various stresses (Song et al., 2015; Zhao et al., 2017; Yang et al., 2019). Both ZntR and Zur, two zinc responsive regulators, are also involved in T6SS-4 regulation by directly binding to its promoter region (Wang et al., 2017; Cai et al., 2021).

The type VI secretion system-4 dependent zinc transport also plays a crucial role in the interactions of pathogenic *Y. pseudotuberculosis* with its mammalian host, as *Y. pseudotuberculosis* T6SS-4 mutants are attenuated in virulence against mice. Especially, mutation of T6SS-4 or *yezP* together with *znuCB* [a classic zinc transporter known to combat host nutritional immunity (Hood et al., 2012; Liu et al., 2012)] resulted in mutants that almost completely lost the virulence against mice, suggesting the importance of T6SS-4 the resistance to host nutritional immunity (Wang et al., 2015). This finding revealed a new mechanism of T6SS in pathogenesis. Further studies on *Burkholderia thailandensis* have revealed the mechanism of zinc ions transport across the membrane through T6SS (Si et al., 2017a). The T6SS-4 in *B. thailandensis* is involved in zinc acquisition via contact-independent secretion of a zinc-scavenging protein, TseZ (BTH_II1884), which cooperates with HmuR, the outer membrane heme transporter for zinc acquisition. T6SS secreted TseZ directly binds zinc ions and interacts with the heme transporter HmuR to transport zinc across the outer membrane. HmuR is a redox-regulated dual functional transporter. Under normal conditions, HmuR is used mainly for the transport of heme-iron; HmuR switches to transport of zinc upon sensing extracellular oxidative stress. Under mild oxidative stress condition, HmuR-mediated zinc transport alone is sufficient to maintain intracellular redox homeostasis. In contrast, under severe oxidative stress challenge, T6SS-4 is fully induced and secretes the proteinaceous zincophore TseZ to enhance the efficiency of HmuR-mediated zinc transport (Si et al., 2017a).

In *Burkholderia pseudomallei*, the T6SS-2 gene cluster also encodes a zinc binding protein (TseZ). TonB-dependent transporters that interact with TseZ and actively transport Zn^2+^ across the outer membrane have also been identified as BhuR (DeShazer, 2019).

### Manganese

Manganese is also an essential micronutrient for many cellular components or processes, such as lipid, protein, carbohydrate metabolism, transcriptional regulation, and resistance to oxidative stress (Kehres and Maguire, 2003). Manganese plays a crucial role in bacterial iron homeostasis and protection against oxidative damage (Puri et al., 2010). Two manganese ions, Mn^2+^ and Mn^3+^, are found in most organisms. In contrast to Fe^2+^, free Mn^2+^ is not toxic in a biological environment; thus, it can replace the more reactive Fe^2+^ in Fe^2+^-containing proteins, reducing oxidative damage to these proteins (Hood and Skaar, 2012). Manganese can also enhance oxidative stress resistance by serving as a cofactor for ROS-detoxifying enzymes, such as SodA and KatN, or through the formation of non-proteinaceous manganese antioxidants (Aguirre and Culotta, 2012; Barnese et al., 2012). Two major transporters import extracellular manganese across the cytoplasmic membrane: a proton-dependent Nramp-related transport system (MntH) and an ATP-binding cassette transporter (SitABCD and YfeABCD) (Goswami et al., 2001; Forbes and Gros, 2003).

The *B. thailandensis* T6SS-4 plays an important role in survival under oxidative stress by uptake Mn^2+^ through secreting TseM (BTH_II1883). TseM, a T6SS-4-dependent Mn^2+^-binding effector, is involved in the intracellular accumulation of manganese (Mn^2+^) under oxidative stress, and an Mn^2+^-specific TonB-dependent outer membrane transporter MnoT, has been shown to be its interacting partner (Si et al., 2017b). Under high Mn^2+^ conditions, passive diffusion of Mn^2+^ through porins fulfills cellular Mn^2+^ requirements. Low Mn^2+^ triggers the induction of the TonB-dependent outer membrane transporter MnoT for the active transport of Mn^2+^ across the outer membrane. T6SS-4 expression is activated by the conserved oxidative stress regulator OxyR. Activated T6SS-4 secretes TseM into the extracellular milieu to scavenge Mn^2+^ and delivers its Mn^2+^ load to MnoT via direct interaction. The T6SS-MnoT mediated active Mn^2+^ transport system also participates in the interbacterial competition and bacterial virulence. The T6SS-4 provides growth advantage in nutrient-limited environments and is critical for virulence in *Galleria mellonella* larvae (Si et al., 2017b). Similarly, a Mn^2+^-binding effector (TseM) secreted by T6SS-2, together with its transmembrane transporter MnoT, was used to maintain redox homeostasis via Mn^2+^ acquisition in the *B. pseudomallei* complex (DeShazer, 2019). Recently, *Y. pseudotuberculosis* T6SS-4 was also found to secret a Mn^2+^-binding micropeptide, TssS, for Mn^2+^ acquisition and oxidative stress resistance. Remarkably, TssS was revealed to be delivered into host cells to inhibit the STING-mediated innate immune response by sequestering Mn^2+^. This finding provides a new perspective on the role of the T6SS in pathogenesis (Zhu et al., 2021).

### Iron

Iron is an essential nutrient for living organisms by acting as a cofactor for a large number of enzymes and regulatory proteins. Although iron is abundant in the Earth’s crust, the bioavailability iron is severely restricted due to extremely low solubility under aerobic conditions (Schaible and Kaufmann, 2004; Miethke and Marahiel, 2007). To acquire sufficient iron for growth, bacteria have evolved several strategies, including import of ferrous iron by ATP- or GTP-dependent inner membrane transporters and TonB-ExbB-ExbD-dependent transport of ferric-siderophores, transferrin, haem, or haem-bound proteins through specific outer membrane receptors (Braun, 2001; Hood and Skaar, 2012).

*Pseudomonas aeruginosa* competes for iron by producing the high affinity siderophores pyoverdine and pyochelin, as well as hemophores, and it can also import xenosiderophores released by other bacteria (Cornelis, 2010). As a *P. aeruginosa* mutant lacking three known iron acquisition systems (PAΔ3Fe) retains the ability to grow in an iron deficient media, a novel iron acquisition pathway coupling the H3-T6SS effector TseF (PA2374), *Pseudomonas* quinolone signal (PQS, 2-heptyl-3-hydroxy-4-quinolone), outer membrane vesicles (OMVs), and the outer membrane receptors FptA and OprF was identified (Lin et al., 2017). TseF does not bind iron, but it interacts with the iron chelating PQS with a high affinity. The PQS molecule has been long known to bind iron with a high affinity but the physiological role of such binding remains unknown (Bredenbruch et al., 2006; Diggle et al., 2006). TseF engages siderophore receptor FptA and the porin OprF for iron acquisition (Nissen-Meyer et al., 1992). Consistent with the biochemical results, both FptA and OprF are required for TseF-mediated iron acquisition. Like the hydrophobic PQS, TseF is incorporated into outer membrane vesicles (OMVs), which have been suggested to play a role in iron acquisition in *P. aeruginosa* by unknown mechanism (Kulp and Kuehn, 2010). The T6SS substrate TseF integrates several molecules previously known to be involved in iron acquisition to transport iron to the cell. The *tseF* gene is present in many bacteria, suggesting wide use of this iron acquisition mechanism. The H3-T6SSpromoters and *tseF* expression for iron acquisition are commonly repressed by the ferric uptake regulator (Fur) for intracellular iron homeostasis (Lin et al., 2017). In *Cupriavidus necator*, T6SS1 secreted TeoL preferentially in association with OMVs through interactions with LPS, which enables bacterial cells to recruit OMVs derived from different species and confers advantages to bacterial cells for iron acquisition (Li et al., 2021).

An iron chelator, pyoverdine, secreted by *Pseudomonas taiwanensis*, can inhibit the growth of the rice bacterial blight pathogen *Xanthomonas oryzae* pv. *oryzae (Xoo*). T6SS is involved in the secretion of the endogenous iron chelator pyoverdine; however, the mechanism is unknown (Chen et al., 2016). Notably, the regulation of T6SS by Fur or iron has also been reported in *Escherichia coli* (Brunet et al., 2011), *Edwardsiella tarda* (Chakraborty et al., 2011), *Burkholderia mallei*, and *B. pseudomallei* (Burtnick and Brett, 2013), implicating the possible roles of these T6SSs in iron acquisition.

### Copper

As one of the most stable divalent transition metals, cupric copper (Cu^2+^) displays a high affinity for metalloproteins (Waldron and Robinson, 2009). Copper is a catalyzer for electron transfer reactions in bacteria and a cofactor of copper-detoxifying enzymes (Dupont et al., 2011; Hodgkinson and Petris, 2012). Because copper is toxic, intracellular copper levels must be tightly controlled to ensure the homeostasis required for cuproprotein synthesis and prevent toxic effects (Argüello et al., 2013). ComC in *E. coli* represses copper uptake and thus plays an important role in copper homeostasis, and its homologs have been found in many gram-negative bacteria (Rademacher and Masepohl, 2012). For the import of copper, a few cytoplasmic Cu^2+^-sensing transcriptional regulators (CueR, CsoR, and CopY) (Strausak and Solioz, 1997; Outten et al., 2000; Liu et al., 2007) and periplasmic Cu^2+^-sensing two-component systems (CopR/S, CusR/S, and PcoR/S) (Rensing and Grass, 2003; Teitzel et al., 2006) have been found to play important roles. However, copper efflux in pathogenic enterobacteria is more crucial than copper uptake (Nies and Herzberg, 2013). The inner membrane heavy metal pumps (transmembrane P1B-type ATPases) in many gram-negative bacteria are responsible for the exportation of cytoplasmic copper to the periplasm (Klein and Lewinson, 2011).

In *P. aeruginosa*, azurin (Azu) has a high affinity for oxidized Cu^2+^-bound proteins (Nar et al., 1992; Zhang and Rainey, 2008). Based on an analysis of the *P*. *aeruginosa* H2-T6SS-dependent secretomes, Azu was characterized as an H2-T6SS-dependent copper (Cu^2+^)-binding effector. OprC, a Cu^2+^-specific TonB-dependent outer membrane transporter, has been identified as an Azu-interacting partner. Both Azu and OprC are directly regulated by the transcriptional regulator CueR and are induced by low Cu^2+^concentrations (Han et al., 2019).

*Pseudomonas aeruginosa* possesses three T6SS loci: H1-, H2-, and H3-T6SSs that provide a fitness advantage in bacterial community competition by delivering toxins to target cells (Mougous et al., 2006; Russell et al., 2011). The identified T6SS-dependent antibacterial toxin effectors include Tse1-Tse3, PldA, TplE, and PldB (Russell et al., 2011; Jiang et al., 2014; Sana et al., 2015). T6SS-mediated Cu acquisition also provides a growth advantage in bacterial competition, indicating the critical role of the Azu-OprC-mediated Cu^2+^ transport system (Han et al., 2019). Like VgrG2b that is secreted by H2-T6SS and shows an anti-eukaryotic function, the H2-T6SS-dependent Cu^2+^ transport system is important for bacterial virulence in the blood and lungs of infected mice (Sana et al., 2015; Han et al., 2019).

### Molybdenum

Molybdenum is a trace metal element for nitrate metabolism in many bacteria and exists in the form of its oxyanion, molybdate (MoO_4_^2–^) under natural conditions (Grunden and Shanmugam, 1997). Bacteria acquire molybdate mainly through the high-affinity ATP-binding cassette permease ModABC (Pederick et al., 2014) and non-specific anion importers (Self et al., 2001). The imported MoO_4_^2–^often becomes a part of the Manganese chelating protein molecule to form a molybdenum cofactor, participating in the activity of molybdo-enzymes (Kraft et al., 2011).

In *P*. *aeruginosa*, the H2-T6SS secreted ModA has been identified as a molybdate-binding protein and mediated molybdate acquisition. Moreover, a ModA partner that participates in molybdate transport has also been identified as IcmP that is an insulin-cleaving metalloproteinase outer membrane protein (Wang et al., 2021). The T6SS-ModA-IcmP system contributes to bacterial virulence and participates in bacterial competition under anaerobic conditions. Studies have shown that the molybdenum homeostasis of *P. aeruginosa* PA1006 is necessary for nitrate utilization, biofilm formation, and virulence (Filiatrault et al., 2013; Tombline et al., 2013). In a mouse model of acute pneumonia, the *P*. *aeruginosa* Δ*clpV2*, Δ*modA*, and Δ*anr* mutants exhibited attenuated virulence, indicating that the H2-T6SS-mediated molybdate transport system contributes to the resistance to host nutritional immunity (Wang et al., 2021). Like the previously discovered two H1-T6SS effectors Tse1 and Tse3, which can hydrolyze peptidoglycan and provide a competitive fitness advantage (Russell et al., 2011), H2-T6SS improves bacterial competition by promoting molybdate (MoO_4_^2–^) acquisition under anaerobic conditions (Wang et al., 2021). As a transcriptional regulator, Anr can activate H2-T6SS expression under anaerobic conditions (Wang et al., 2021). Anr in *P*. *aeruginosa* controls the switch from aerobic to anaerobic growth and plays a pivotal role in adapting to microaerobic or anoxic conditions (Ugidos et al., 2008; Tata et al., 2017).

## Conclusion

All organisms keep metal homeostasis for physiological demands by sensing small fluctuations in metal levels (Porcheron et al., 2013). Bacteria have developed complex transport systems for each metal whose expression is coordinated by their corresponding regulators (such as Fur, MntR, CueR, and Zur, etc.) (Wakeman and Skaar, 2012). T6SS was a newly found device for bacteria to acquire metal ions, expanding our understanding on sophistication of bacterial metal ion acquisition systems. T6SS participation in metal ion uptake, which assists the bacterial low- and high-affinity transport systems to scavenge from the environment essential metals in chelated or free forms. In addition, the metal ion transport function of T6SS is usually involved in multiple biological processes and is crucial for bacterial survival and host colonization. Traditionally, T6SS is recognized as a contact-dependent molecular machinery. Recent studies revealed that T6SSs play crucial roles in shaping the composition of a microbial population in hosts or environmental niches, either by directly killing competing cells via contact-dependent (Russell et al., 2011) and contact-independent (Song et al., 2021) translocation of toxins, or by competing for essential nutrients via contact-independent secretion of metal ion binding effectors. For example, the T6SS-HmuR-mediated active zinc transport system is involved in a contact-independent bacteria-bacteria competition for nutrients (Si et al., 2017a,b; Han et al., 2019; Wang et al., 2021).

**FIGURE 1:**
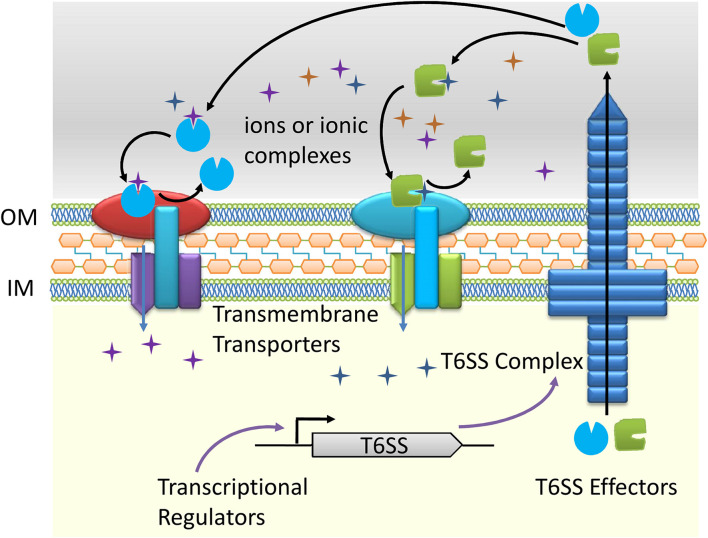
Schematic diagram of the type VI secretion system (T6SS) dependent ions transport.

A schematic diagram was used to show the process of metal ions transport through T6SS (Figure 1). Briefly, the T6SS dependent effectors bind to specific metal ions or ionic complexes in the environment. Accompanied by a transmembrane ion transporter, the corresponding ions are transferred to the cell. The ions transport process is collaboratively fulfilled by the T6SS effectors and their transmembrane partners. It is worth mentioning that the metal ions transporting function of T6SS is often activated under special circumstances, such as low ions concentrations or environmental stresses and is regulated by multiple transcriptional regulators. So far, it is unknown whether T6SS correlates with metal ions efflux. We believe that ions transport through T6SS expands the range of functions associated with this secretory nanomachines and merits additional studies in other bacteria.

## Author Contributions

XY, HL, and YZ collected and assessed the references. XS and HL contributed in the proposal and article layout of the review. XY and XS wrote the manuscript. All authors contributed to the article and approved the submitted version.

## Conflict of Interest

HL and YZ was employed by the company Qingyang Longfeng Sponge City Construction Management & Operation Co., Ltd. The remaining authors declare that the research was conducted in the absence of any commercial or financial relationships that could be construed as a potential conflict of interest.

## Publisher’s Note

All claims expressed in this article are solely those of the authors and do not necessarily represent those of their affiliated organizations, or those of the publisher, the editors and the reviewers. Any product that may be evaluated in this article, or claim that may be made by its manufacturer, is not guaranteed or endorsed by the publisher.

## Abbreviations

T6SS: type VI secretion system
Azu: azurin.
